# Neurogenic rosacea successfully treated with neuromodulators and intense pulsed light^☆^

**DOI:** 10.1016/j.abd.2022.09.016

**Published:** 2023-12-14

**Authors:** Diana Isabel Conde Hurtado, Andrea Paola Céspedes Pérez, Ricardo Flaminio Rojas López

**Affiliations:** DermaHair Center, Floridablanca, Colombia

Dear Editor,

A 36-year-old female patient with two years of erythematous macules localized to the cheeks that later involved the whole face (Fig. 1), associated with intense local burning pain and heat sensation, exacerbated by exposure to ultraviolet radiation, hot temperatures, and strong emotions.

**Figure 1:**
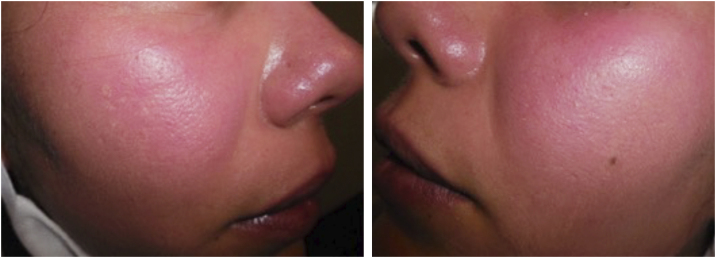
Patient at the time of first consultation with evidence of profuse erythema predominantly on cheeks.

She was diagnosed with erythematotelangiectatic rosacea and received conventional rosacea treatments, such as doxycycline and topical ivermectin, with brief improvement followed by worsening of the symptoms. She also had a history of anxiety and depression.

We rule out other possible causes of *red face syndrome* such as systemic lupus erythematosus, carcinoid syndrome, and pheochromocytoma through laboratory and imaging studies. Given the history of poor response to standard treatments and the concomitance of neurological symptoms, we diagnosed Neurogenic Rosacea (NR). She was treated with pregabalin 150 mg/day and duloxetine 90 mg/day for six months. This significantly reduced pain and burning symptoms. However, the erythema persisted therefore Intense Pulsed Light (IPL) therapy was added (Harmony-Alma Laser®, wavelength of 550‒650 nm, fluence of 10 Joules/cm^2^, pulse width of 12 ms). A small test exposure was made to make sure was adequately tolerated (Fig. 2). She received a monthly session for three months while continuing with pharmacological treatment. After this combined therapy all her signs and symptoms disappeared (Fig. 3) improving her quality of life.

**Figure 2:**
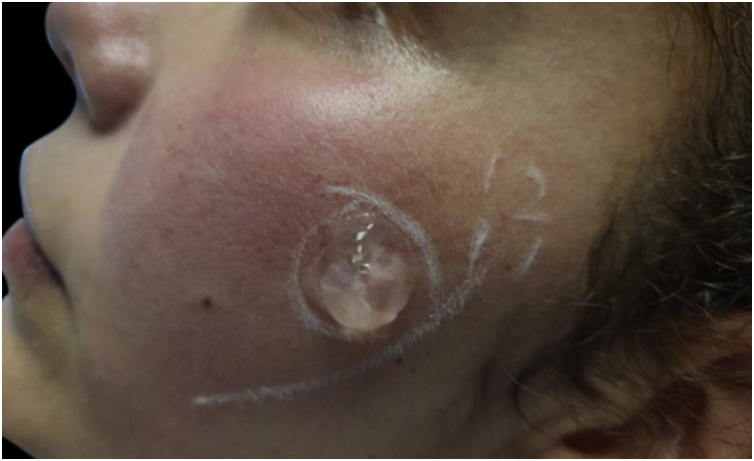
Trial of IPL therapy on a small area of the left cheek, after six months of pharmacotherapy with pregabalin and duloxetine, but persistence of erythema.

**Figure 3:**
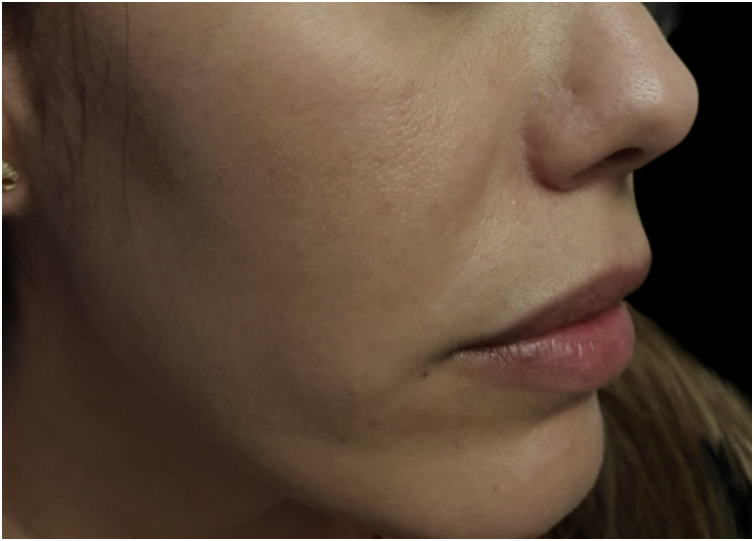
Three months after concomitant use of pharmacotherapy and IPL.

NR develops intense facial redness associated with stinging, burning, and dysesthesias out of proportion to the concomitant flushing or swelling,^1^ and was described by Scharschmidt in 2011.^2^ The degree of erythema is more severe in NR than in other variants, and the pain has been described as stabbing, electric shock-like, or shooting. Triggering factors in NR are similar to other rosacea variants. NR patients have an association with complex regional pain syndrome, essential tremor, anxiety, depression, and obsessive-compulsive disorder.^1^, ^2^

The pathophysiology is still unknown but is thought to be a dysfunctional regulation in the neurovascular system called *neurogenic inflammation*,^1^, ^3^ which is mediated by neuromodulators. The most important are Calcitonin Gene-Related Protein (CGRP) and Substance P (SP). They act on the endothelial cells and smooth muscle cells leading to vascular changes. SP induces edema and neovascularization. CGRP is a potent microvascular vasodilator that worsens local inflammation.^3^

The response of NR is poor to conventional therapies.^4^ Drugs that attenuate neurotransmitter release could be effective (pregabalin, gabapentin, antidepressants, memantine, and duloxetine).^1^ Pregabalin modulates the release of SP and CGRP. Duloxetine has an anti-inflammatory and immune-modulatory effect.^5^ Both drugs helped with her anxiety and depression symptoms as well as the somatic symptoms.

Light therapies, like IPL, are successful for rosacea. IPL uses photothermolysis to destroy blood vessels reducing facial erythema. The fluence, pulse width, and inter-pulse interval considerations will depend on the patient's phototype, the severity of the condition, and tolerance to treatment, and is adjustable according to the physician’s criteria. Light-based interventions should be used with caution because of skin sensitivity.^2^

Other treatments are surgical intervention (sympathectomy), botulinum toxin, and local cold stimuli.^4^

Few NR cases are described, and there still exist gaps in its management. Our patient had a successful response to neuromodulators and IPL without side effects. This combination has not been previously reported.

## Financial support

None declared.

## Authors’ contributions

Andrea Paola Cespedes Pérez: Adequate the study concept and design; writing of the manuscript; research guidance; critical review of the literature; critical review of the manuscript; approval of the final version of the manuscript.

Diana Isabel Conde Hurtado: Adequate the study concept and design; writing of the manuscript; research guidance; critical review of the literature; critical review of the manuscript; approval of the final version of the manuscript.

Ricardo Flaminio Rojas López: Adequate the study concept and design; acquisition of data, analysis of data; intellectual participation in propaedeutic and/or therapeutic conduct of the studied case; writing of the manuscript; critical review of the literature; critical review of the manuscript; approval of the final version of the manuscript.

## Conflicts of interest

Dr. Ricardo Flaminio Rojas is an advisory board member and speaker at Galderma.
